# Ticked Off: A Case of Disappearing Cell Lines

**DOI:** 10.7759/cureus.73844

**Published:** 2024-11-17

**Authors:** Mahnoor Saad, Laila Babar, Sheza Khawaja

**Affiliations:** 1 Internal Medicine, Howard University Hospital, Washington DC, USA; 2 Internal Medicine, Allegheny General Hospital, Pittsburgh, USA; 3 Internal Medicine, Weiss Memorial Hospital, Chicago, USA

**Keywords:** anaplasmosis, cytopenia, doxycycline, tick, tick-borne infections

## Abstract

Human granulocytic anaplasmosis (HGA) is transmitted by the black-legged tick *Ixodes scapularis* and presents with fever, thrombocytopenia, leukocytopenia, and elevated transaminases. If left untreated, HGA can progress to hemophagocytic lymphohistiocytosis (HLH), which can be fatal. Here, we discuss a case of a woman diagnosed with anaplasmosis who was treated promptly.

A 63-year-old female presented with a two-day history of fever and fatigue. She denied any history of hiking but confirmed an avid gardening hobby. She did not report any tick bites or rashes. Upon presentation, she was febrile but hemodynamically stable. Her laboratory results showed elevated liver function tests (LFTs) and bicytopenia, with a white blood cell count (WBC) of 0.77 k/mcL (normal = 4.4-11.3 k/mcL), an absolute neutrophil count (ANC) of 150/µL (normal = >500/µL), and a platelet count of 50 k/mcL (normal = 145-445 k/mcL). Her liver function tests (LFTs) were elevated, including alanine aminotransferase (ALT) of 75 U/L (normal = 0-40 U/L) and aspartate aminotransferase (AST) of 66 U/L (normal = 0-40 U/L). Her lab results had been normal one month prior during routine testing. She was tested for Lyme antibodies, *Ehrlichia* antibodies, and anaplasmosis via a polymerase chain reaction (PCR) test. She was started on doxycycline, and within two days, her counts began to recover. Her Lyme antibody titers and *Ehrlichia* antibody tests were negative, but she tested positive for *Anaplasma phagocytophilum* antibodies, confirming anaplasmosis. She was discharged home, and on follow-up, her WBC had risen to 4.6 k/mcL (normal = 4.4-11.3 k/mcL), and platelets increased to 84 k/mcL (normal = 145-445 k/mcL).

Human granulocytic anaplasmosis is a rickettsial disease that typically presents with symptoms such as fatigue, malaise, fever, thrombocytopenia, and leukopenia. It carries a small but significant risk of progressing to HLH if not treated early in its course. Human granulocytic anaplasmosis has a presentation similar to ehrlichiosis and is differentiated based on PCR testing for deoxyribonucleic acid (DNA); however, since treatment is the same for both diseases, patients are often started on doxycycline before confirmatory test results are available. Physicians working in tick-endemic areas must maintain a high suspicion for rickettsial illnesses, as these patients often present with vague symptoms and can be undiagnosed if not properly evaluated.

## Introduction

Human granulocytic anaplasmosis (HGA) is a tick-borne bacterial illness characterized by fever, thrombocytopenia (low platelets), leukocytopenia (low white cell count), and elevated transaminase levels [1]. First reported as a human disease in 1990, anaplasmosis has since become widely recognized, with the CDC releasing annual epidemiological data and information on its geographical distribution. The disease is caused by the *Anaplasma phagocytophilum* bacterium, a tick-borne pathogen [2]. This bacterium has been known by various names, including *Ehrlichia equi* and *Ehrlichia phagocytophilum*. However, after a taxonomic revision in 2001, it was classified under the *Anaplasma* genus, and the disease name was changed from ehrlichiosis to anaplasmosis [3].

The geographical distribution of anaplasmosis follows that of its vector, the black-legged* Ixodes scapularis* tick, and is primarily concentrated in the upper Midwestern and Northeastern parts of the United States [4]. This geographical spread has been expanding in recent years, as the distribution of* Ixodes scapularis* has steadily increased. This tick also carries *Borrelia burgdorferi*, the causative agent of Lyme disease, and patients may present with co-infections. Approximately 90% of anaplasmosis cases are reported in Vermont, Maine, Rhode Island, Minnesota, Massachusetts, Wisconsin, New Hampshire, and New York [5]. Some cases have also been reported in the Southern states, though this may be due to misdiagnosis as ehrlichiosis, which presents similarly to anaplasmosis.

Human granulocytic anaplasmosis is treated with tetracycline antibiotics, most commonly doxycycline, for a duration of 10 to 14 days [2]. If diagnosed early, the disease is typically benign with a short recovery period. However, if left undiagnosed and untreated, it can progress to hemophagocytic lymphohistiocytosis (HLH) [6] which is a life-threatening condition caused by the excessive activation of the inflammatory system, leading to the hyperproliferation of lymphocytes and macrophages. This results in the release of high levels of cytokines, which contribute to significant morbidity and mortality. Given the increasing prevalence of anaplasmosis and its association with catastrophic complications such as HLH, physicians must include it in their differential diagnosis when working in areas endemic to tick-borne illnesses, regardless of the incidence of anaplasmosis. Here, we present the case of a 63-year-old woman presenting to a tertiary care center in Western Pennsylvania with anaplasmosis.

## Case presentation

A 63-year-old Caucasian female patient presented to the hospital in Pittsburgh in early July with a two-day history of low-grade fever and generalized fatigue. She denied any cough, shortness of breath, dizziness, or gastrointestinal symptoms. She had a history of hypertension and hyperlipidemia and was on optimal medical therapy. On presentation, she was febrile with a low-grade fever of 100.1°F (37.8°C), and all other vital signs were within normal limits. Her lab results were significant for leukopenia, with a white blood cell (WBC) count of 0.77 k/mcL (normal = 4.4-11.3 k/mcL), an absolute neutrophil count (ANC) of 150/µL (normal = >500/µL), a platelet count of 50 k/mcL (normal = 145-445 k/mcL), and mildly elevated aspartate aminotransferase (AST) and alanine aminotransferase (ALT) levels of 66 U/L (normal = 0-40 U/L) and 75 U/L (normal = 0-40 U/L), respectively. She had had a complete blood count (CBC) and complete metabolic panel (CMP) done one month earlier for her annual physical exam, and all values had been within normal limits at that time. 

A computed tomography (CT) scan of her chest, abdomen, and pelvis was performed to investigate potential infectious or hematologic sources and was unremarkable. By the second day, her cell counts were repeated, showing a decline in her WBC count to 0.57 (normal = 4.4-11.3 k/mcL) and a platelet count of 33 (normal = 145-445 k/mcL). Hematology and infectious disease were consulted due to the worsening cell counts, though she had not spiked a fever in the previous 24 hours. A more detailed history was taken, and she informed us that she had spent the last few months in and out of hospitals due to her husband's end-stage cancer and had started gardening to help cope with the stress. She denied any history of hiking and did not recall any tick bites or rashes.

She was initially started on broad-spectrum antibiotics with vancomycin and doxycycline to cover for methicillin-resistant *Staphylococcus aureus* (MRSA) and atypical organisms, as she had been frequently in and out of hospitals for the past three months. Lyme antibody testing was performed and returned negative. A bone marrow biopsy was planned to assess for bone marrow pathology due to the affected cell lines. Ehrlichia and anaplasmosis polymerase chain reaction (PCR) tests were ordered and came back positive for anaplasmosis. Following positive PCR results for anaplasmosis, vancomycin was discontinued, and doxycycline was maintained as the targeted antibiotic therapy.

Outcome

Within two days, her cell counts improved, and the bone marrow biopsy was canceled. Upon discharge, her WBC count was 4.6 k/mcL (normal = 4.4-11.3 k/mcL) and platelets were 84 k/mcL (normal = 145-445 k/mcL). She was advised to follow up with her primary care physician (PCP) in one week for a repeat CBC. One week later, her counts had completely recovered, and her symptoms had resolved. A trend of her laboratory values is seen in Table 1. 

**Table 1:** The Patient's Laboratory Values

Lab Test	Initial Value	Day 2 (After Treatment)	Discharge (Day 4)	1 Week Follow-up
White Blood Cell (WBC) (4.4–11.3 k/mcL)	0.77	0.57	4.6	6
Absolute Neutrophil Count (ANC) (>500 /µL)	150	120	>500	>500
Platelet Count (145–445 k/mcL)	50	33	84	145
Aspartate Aminotransferase (0–40 U/L)	66	70	50	42
Alanine Aminotransferase (0–40 U/L)	75	92	62	48

## Discussion

Since its recognition in the 1990s, the incidence of HGA has been steadily increasing. In 2000, there were 348 reported cases of HGA in the United States, which increased to over 4,151 cases by 2016 [4]. Nearly ninety percent of the reported cases of HGA occur in eight states (Vermont, Maine, Rhode Island, Minnesota, Massachusetts, Wisconsin, New Hampshire, and New York). However, cases are also reported annually in other areas where the *Ixodes scapularis* tick is found. Our patient was from Western Pennsylvania, an area endemic to Lyme disease and home to a large population of *Ixodes scapularis* ticks. These ticks are found in all 67 counties in Pennsylvania [4] and are the primary vectors for *Anaplasma phagocytophilum* as well as *Borrelia burgdorferi*. In 2015, it was estimated that 3.3% of these ticks carried *Anaplasma phagocytophilum* in Pennsylvania, with approximately three to 25 cases of anaplasmosis per million people reported annually [7]. Given the rising incidence of anaplasmosis and the expanding range of the tick, physicians in endemic areas should be encouraged to familiarize themselves with these rickettsial diseases, their presentations, and treatment options.

Human granulocytic anaplasmosis often presents with vague, generalized symptoms that can be overlooked by physicians or may not even be mentioned by patients. The disease can have various presentations, but it is most commonly characterized by fever, generalized body aches, and respiratory or gastrointestinal symptoms. Patients often report a history of a tick bite or time spent outdoors in areas with a high tick population. Our patient presented with fever and myalgia but denied any history of a tick bite. Upon further inquiry, she mentioned a new gardening hobby, which may have exposed her to ticks and increased her risk of developing the disease. Her lab results were concerning, with elevated liver transaminases and low cell counts. Combining her history, residence in an endemic area, and abnormal lab results raised our suspicion for anaplasmosis.

Although our patient exhibited characteristic lab abnormalities and disease symptoms, her diagnosis was initially delayed due to an atypical exposure history and the absence of a reported tick bite or rash. A high index of suspicion is crucial for early diagnosis. Polymerase chain reaction testing for anaplasma can take 24 to 48 hours to return results, so patients should be started on treatment before a positive result is obtained. Delayed treatment can lead to catastrophic sequelae, particularly in vulnerable populations such as immunocompromised individuals, patients with HIV/AIDS, the elderly, and organ transplant recipients [2-3]. Severe respiratory distress, meningitis, sepsis, or HLH can develop if treatment is delayed [6].

Lab tests to confirm *Anaplasma phagocytophilum* infection include PCR, peripheral blood smear, and immunohistochemistry. Polymerase chain reaction testing is the most commonly used method, as it has high sensitivity and specificity and provides rapid results. A peripheral blood smear can be drawn before or within 24 to 48 hours of starting antibiotics. Approximately one-quarter of infected patients will have morulae visible in their blood smear [6]. Immunohistochemistry is used in autopsy settings to identify the cause of death in patients who do not survive the infection. Polymerase chain reaction testing also helps differentiate anaplasmosis from ehrlichiosis, as both diseases have similar presentations. The treatment for both is the same (doxycycline), but knowing the exact cause of the illness is helpful for accurate reporting and epidemiological records.

With the increasing rates of infection over the years [8-10], it is paramount that the treatment of anaplasmosis be started if the index of suspicion is high so as to avoid devastating consequences.

## Conclusions

Physicians working in tick-endemic areas should have a high index of suspicion for all tick-borne illnesses, even when the incidence of a particular disease is low. These infections are easily treatable if caught at an appropriate time; however, they can have severe consequences if left untreated or diagnosed late. Clinicians should initiate treatment promptly if anaplasmosis is suspected based on symptoms and patient history without awaiting confirmatory PCR results, as delays in antibiotic administration can increase the risk of severe complications.
